# Prophylaxis and management of thromboembolism in pnh patients: an italian survey

**DOI:** 10.1007/s00277-026-07045-6

**Published:** 2026-07-15

**Authors:** Anna Paola Iori, Rosario Notaro, Martina Salvatori, Camilla Frieri, Eros Di Bona, Antonio De Vivo, Simona Sica, Andrea Patriarca, Francesco Lanza, Alessandra Ricco, Fabio Giglio, Antonella Sau, Eloise Beggiato, Simona Raso, Alessandro Costa, Amalia Figuera, Maria Bruna Greve, Filippo Achille Brioschi, Marica Laurino, Giulio Giordano, Elisabetta Metafuni, Francesca D’adamo, Teresa Maria Santeramo, Angela Amendola, Giorgia Mancini, Bruno Fattizzo

**Affiliations:** 1 Department of Oncology and Hemato-Oncology, University of Milan, Milan, Italy; 2 Fondazione IRCCS Ca’Granda Ospedale Maggiore Policlinico, Milan, Italy; 3 AOU Policlinico Umberto I, UOC Ematologia, Sapienza University of Rome, Rome, Italy; 4 SC Core Research Laboratory, Istituto per lo Studio, la Prevenzione e la Rete Oncologica -ISPRO – PNH, Florence, Italy; 5 SOD Ematologia, Azienda Ospedaliero-Universitaria Careggi, Florence, Italy; 6 Department of Translational and Precision Medicine, Hematology, Sapienza University of Rome, Rome, Italy; 7 AORN San Giuseppe Moscati, Avellino, Italy; 8 UOC Oncoematologia, Pedemontana, Bassano del Grappa, AULSS7 Italy; 9 Hematology Unit, Department of Experimental, Diagnostic and Specialty Medicine (DIMES), S. Orsola-Malpighi University Hospital, University of Bologna, Bologna, Italy; 10 Dipartimento di Scienze di laboratorio ed Ematologiche. Fondazione, Policlinico Universitario A Gemelli IRCCS, Rome, Italy; 11 Hematology Unit, Department of Translational Medicine, University of Eastern Piedmont and AOU Maggiore della Carità, Novara, Italy; 12 Hematology Unit, Ravenna Hospital, University of Bologna, Ravenna, Italy; 13 Unità Operativa di Ematologia con Trapianto, Azienda Ospedaliera Universitaria Consorziale Policlinico di Bari, Bari, Italy; 14 UO Ematologia e Trapianto di Midollo osseo, IRCCS Ospedale San Raffaele, Milano, Italy; 15 U.O.C. Ematologia - P.O. Spirito Santo, Pescara, Italy; 16 University Hospital City of Health and Science of Turin - Hospital Molinette, Oncology and Hematology, Turin, Italy; 17 Department of Hematology and Rare Diseases, Azienda Ospedaliera Ospedali Riuniti Villa Sofia-Cervello, Palermo, Italy; 18 Hematology Unit, Department of Medical Sciences and Public Health, Businco Hospital, University of Cagliari, Cagliari, Italy; 19 Division of Hematology, AOU. Policlinico G. Rodolico-S. Marco, Catania, Italy; 20 Clinical of Haematology Grande Ospedale Metropolitano di Reggio Calabria, Reggio Calabria, Italy; 21 Fondazione IRCCS San Gerardo dei Tintori, Monza, Italy; 22 Clinica Ematologica e C.T.A. Monza, Monza, Italy; 23 Hematology and Cellular Therapy Unit, IRCCS Ospedale Policlinico San Martino, Genoa, Italy; 24 Unità Operativa Complessa, Medicina Servizio e Ambulatorio di Ematologia Ospedale di Riferimento Regionale Antonio Cardarelli, Campobasso, Italy; 25 Hematology and Hematopoietic Stem Cell Transplant Center, San Salvatore Hospital, Pesaro, Italy; 26 Division of Hematology, Monsignor Raffaele Dimiccoli Hospital Barletta, Barletta, Italy; 27 UOC Ematologia con Centro trapianto di Midollo, Cellule staminali e terapie cellulari AOR SAN CARLO, Potenza, Italy; 28 Clinic of Hematology Azienda Ospedaliero Universitaria delle Marche, Ancona, Italy; 29 Haematology AOU Policlinico Umberto I, Sapienza University of Rome, Rome, Italy

**Keywords:** Paroxysmal nocturnal hemoglobinuria, Venous thromboembolism, Complement inhibition, Antithrombotic prophylaxis

## Abstract

Paroxysmal nocturnal hemoglobinuria (PNH) is a rare clonal hematopoietic stem cell disorder characterized by uncontrolled complement-mediated intravascular hemolysis and a disproportionately high thromboembolic risk. Although terminal complement inhibition (CIT) has substantially modified the disease course, reducing thromboembolic events (TE), the residual risk remains elevated and evidence-based anticoagulation guidelines are lacking. To characterize real- world practice, we conducted a nationwide, cross-sectional survey involving 24 Italian Centers caring for 291 patients, evaluating strategies for primary antithrombotic prophylaxis (PAP), secondary prophylaxis (SAP), and management of breakthrough hemolysis (BTH). Marked heterogeneity emerged. PAP prior to CIT was routinely employed in only one-third of Centers, whereas an equal proportion did not use it; the remainder adopted risk-based approaches integrating thrombophilia traits or clone size. SAP was universally administered after TE, with 75% of Centers maintaining indefinite anticoagulation regardless of CIT response. Anticoagulation during BTH was likewise variable, with LMWH as the preferred agent and duration largely dictated by biochemical resolution. These findings demonstrate substantial inter-center variability, underscoring the lack of harmonized national frameworks. The results highlight the need for structured expert consensus and standardized clinical algorithms to optimize thrombosis prevention and management in PNH in the era of complement inhibition.

## Introduction

Paroxysmal nocturnal hemoglobinuria (PNH) is a rare, acquired, clonal, non-neoplastic hematopoietic stem cell disorder characterized by complement-mediated intravascular hemolysis, bone marrow failure, and increased risk of thromboembolic events (TE) [1–3]. Before the use of Complement inhibition therapy (CIT), thrombosis was the leading cause of morbidity and mortality in affected patients [4], prompting PNH to be defined as “the most vicious acquired thrombophilic state known in medicine” [5]. The pathogenesis of thrombosis in PNH is multifactorial, involving several not mutually exclusive mechanisms, including complement-mediated platelets and coagulation activation, release of prothrombotic factors secondary to hemolysis, impaired fibrinolytic activity, and endothelial dysfunction [6].

Indeed, in the pre-complement inhibition therapy (CIT) era, the lifetime risk of thrombosis in individuals with PNH was estimated to range between 40% and 50% [7–10].

Thrombotic events accounted for up to 67% of PNH-related deaths [4, 11, 12] and patients presenting with thrombosis at diagnosis had a 4-year overall survival rate of only 40% [13].

Furthermore, thrombotic episodes in PNH were often unpredictable, sometimes presenting as catastrophic events (e.g., those involving abdominal and cerebral veins at onset) [6, 14, 15], and could even arise in patients receiving adequate anticoagulation therapy [6].

The advent of CIT has significantly transformed the natural history of PNH by effectively controlling hemolysis, and reducing the risk of TE from 7.37 events/100 patient-years prior to eculizumab treatment to 1.07 events/100 patient-years with CIT (*P* < .001) [7–10, 16]. However, considering that the risk of thromboembolic events in the general population is estimated at only 1 to 2 per 1,000 person-years in Western countries, the residual risk in PNH patients remains significantly elevated even during CIT. Thus, managing thrombosis remains a major clinical challenge, largely due to the lack of standardized guidelines [16–21]. Although direct oral anticoagulants (DOACs) have emerged as a potential alternative to vitamin K antagonists (VKAs) in several thromboembolic conditions [19, 21], data on their safety and efficacy in PNH remain limited, leaving a significant gap in evidence-based clinical decision-making [22–25].

Therefore, despite the evolving therapeutic landscapes—marked by the integration of complement inhibitors and novel anticoagulant agents—significant unmet clinical needs persist regarding the optimal management of thrombotic risk in PNH.

This study aimed to evaluate current practices and institutional policies concerning the prophylaxis and management of thrombotic complications in patients with PNH across Italian Centers. By mapping the national approach to thrombosis in PNH, the study seeks to identify shared practices and existing disparities, thereby providing a foundation for the development of standardized and harmonized clinical strategies.

## Methods

A cross-sectional survey was conducted between September 2024 and January 2025 to assess thrombosis management practices among Italian Centers that follow and treat PNH patients, provided that at least one patient per center was treated with a complement inhibitor.

The structured questionnaire comprised the following domains (Table 1):

**Table 1:** Survey questionnaire. BTH: breakthrough hemolysis

Item 1: Primary Phophylaxis
• Do you perform thrombophilia screening at diagnosis of PNH?
• Is anticoagulant prophylaxis administered to PNH patients who are not receiving complement inhibitors or are awaiting treatment initiation, in the absence of a history of thrombosis?
• If yes, in which patients?
• If yes, which drugs do you use?
• Does your center discontinue primary anticoagulant therapy in PNH patients undergoing complement inhibitor treatment?
• If yes, in which cases?
ITEM 2. Secondary Prophylaxis
• Which anticoagulant drugs are used for secondary prophylaxis?
• Does your Center discontinue secondary anticoagulant prophylaxis
• If yes in which patients
ITEM 3. Anticoagulation and BTH
• Do you administer anticoagulant therapy for prophylaxis in patients with BTH?
• If yes, in which cases?
• If yes, which anticoagulant drugs are used?
• If yes, for how long?

- Indication of primary anti-thrombotic prophylaxis (PAP) in patients awaiting initiation of CIT due to logistical delays in drug procurement, the completion of mandatory vaccination protocols, or patient-driven factors, including requests for a second clinical opinion.
- Indication of secondary anti-thrombotic prophylaxis (SAP) and discontinuation of SAP therapy during CIT in patients who experienced thrombotic events before CIT.
- Indication of PAP in patients on CIT, choice of anticoagulants class and duration of PAP.
- Indication of PAP in the event of breakthrough hemolysis (BTH), choice of anticoagulants class and duration of PAP post-BTH.

### Operational definitions

- Primary anti-thrombotic prophylaxis (PAP) is defined as the preventive use of medications to reduce the risk of blood clot formation in patients who have not yet experienced a thrombotic event, but who are considered at increased risk due to hematologic features of PNH or coexisting thrombotic risks.
- Secondary anti-thrombotic prophylaxis (SAP) refers to the ongoing use of medications or interventions to prevent the recurrence of thrombotic events in PNH patients who have already experienced a thrombotic episode.
- Breakthrough hemolysis (BTH) is defined as the occurrence of any level of intravascular hemolysis, despite ongoing treatment with CIT. Clinically significant BTH is the occurrence of intravascular hemolysis associated with significantly increased lactate dehydrogenase (LDH) levels (≥ 2 ×basal value) and falling hemoglobin levels (> 2 gr/dl) [23].

## Results

**Fig. 1:**
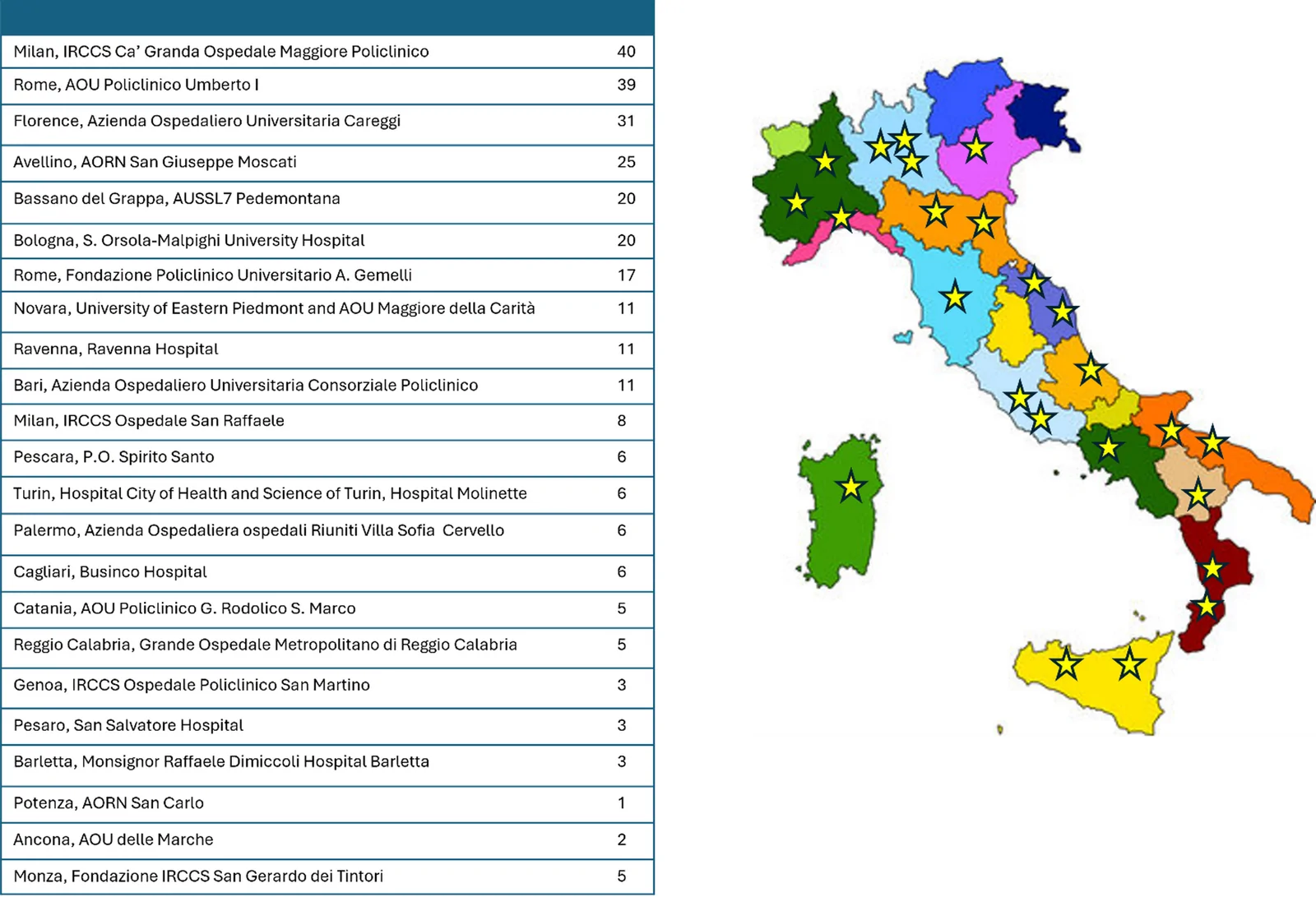
Italian PNH centers and number of patients for each center

Overall, 24 Centers participated in the survey, collectively managing 291 PNH patients. The median number of patients per center was 6 (range, 1–40) (Fig. 1).

### Antithrombotic prophylaxis in patients before CIT

**Fig. 2:**
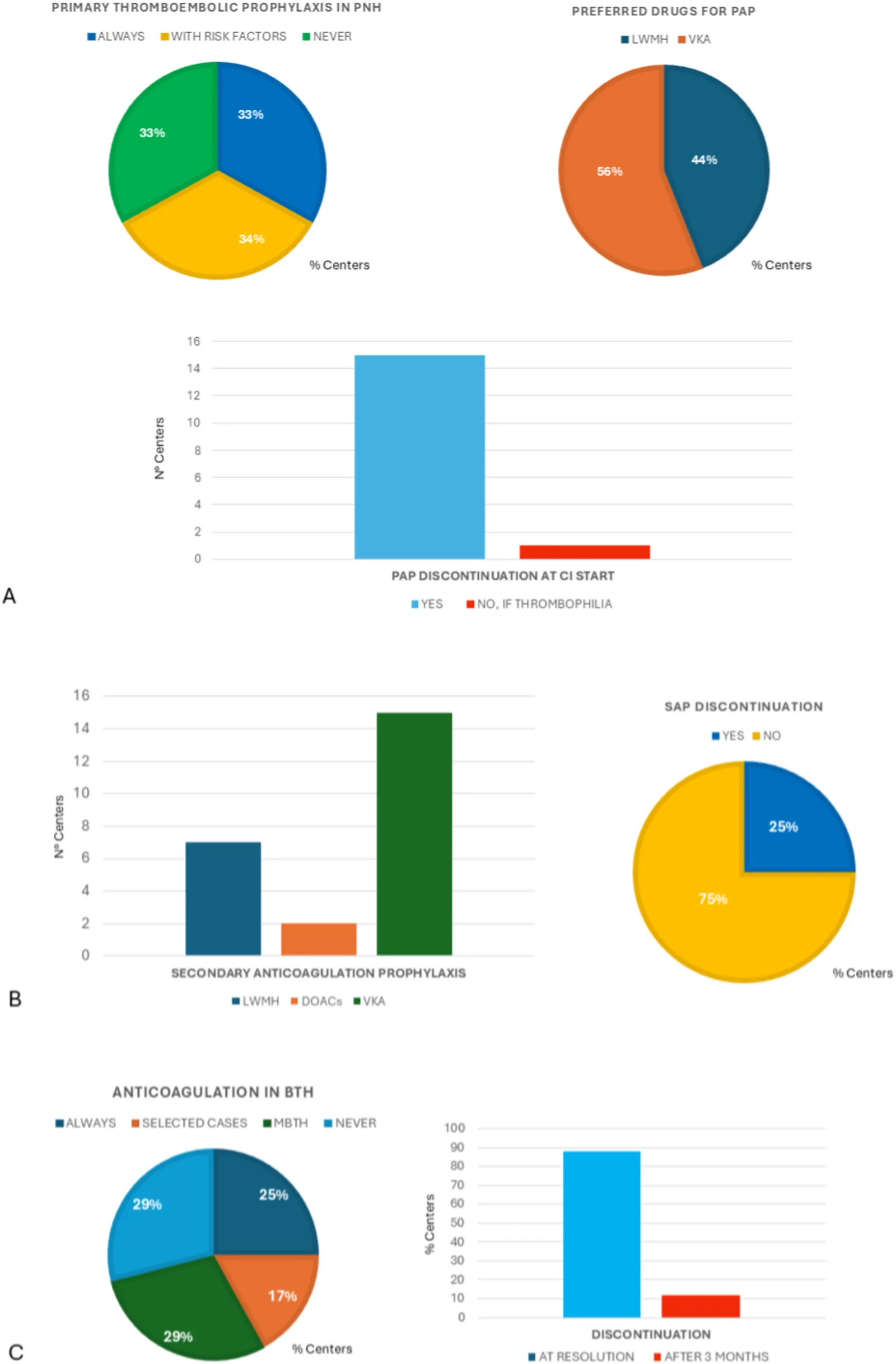
Survey answers. **A**: Antithrombotic prophylaxis in patients before Complement inhibition therapy. **B**: Secondary antithrombotic prophylaxis in patients receiving Complement inhibition therapy. **C**: Antithrombotic prophylaxis in the setting of breakthrough hemolysis. PAP: primary anti-coagulant prophylaxis; LMWH: low molecular weight heparin; DOACs: direct oral anti-coagulants; VKA: vitamin k antagonists; CI: complement inhibitors; SAP: secondary anticoagulant prophylaxis; BTH: breakthrough hemolysis; MBTH: major breakthrough hemolysis

Regarding the use of Primary Anti-thrombotic Prophylaxis (PAP), practices were divided among the surveyed centers (Fig. 2A):

- Eight Centers (33%) reported never using PAP. Eight Centers (33%) reported systematic use of PAP regardless of additional thrombotic risk factors; 6 Centers (25%) reported prescribing PAP only in patients with additional thrombotic risk factors, based on thrombophilia screening results; 2 Centers (9%) utilized PAP based on PNH neutrophils population size (e.g., PNH neutrophils > 50% or PNH neutrophils > 10% associated with positive thrombophilia tests).
- Among the 16 Centers that reported using PAP, VKA was the preferred choice in 9 Centers, and LMWH was preferred in 7 Centers.
- PAP was always discontinued after the initiation of CIT in 15 Centers (93%). One center (7%) reported continuing PAP indefinitely in patients with underlying thrombophilia.
- All 24 Centers reported using SAP in patients with a prior history of TE. The preferred anticoagulants for SAP included VKAs (15 Centers), LMWH (5 Centers), and DOACs (2 Centers). Three Centers reported using VKAs and LMWH interchangeably.

In the case of thrombocytopenia all Centers reported the use of heparin as anticoagulation therapy.

### Antithrombotic prophylaxis in patients receiving CIT

- All Centers reported that anticoagulant prophylaxis was not used after starting CIT in the absence of a history of TE, or of additional thrombotic risk factors identified by thrombophilia screening.
- All Centers reported the use of SAP in patients with documented TE prior CIT. Despite concurrent CIT, 18 centers continue SAP indefinitely. In contrast, the remaining centers consider discontinuation under specific clinical conditions: two centers withdraw SAP following radiologically (ultrasound, CT, MRI) confirmed resolution of thrombosis and two years of event-free (EF) follow-up; two centers limit discontinuation to cases of superficial thrombosis; and two centers discontinue SAP provided there is no history of breakthrough hemolysis (BTH) or thrombosis at atypical sites (Fig. 2B).

### Antithrombotic prophylaxis in the setting of breakthrough hemolysis (BTH)

- 7 Centers (29%) reported never using PAP during episodes of BTH. 6 Centers (25%) reported the use of PAP in all BTH episodes and 7 Centers (29%) reported using PAP only in clinically significant BTH episodes. The remaining 4 Centers (17%) utilized PAP only in BTH episodes associated with additional thrombotic risks (i.e., infections or prolonged bed rest).
- LMWH was the sole anticoagulant reported for PAP during BTH, with dosing consistently adjusted based on the patient’s platelet count. Among the 17 Centers that reported the use of PAP during BTH, anticoagulation was discontinued once hemolytic parameters returned to baseline in 15 Centers (88%), while 2 Centers (12%) continued anticoagulation for 3 months following the resolution of BTH (Fig. 2C).

## Discussion

Despite the evolving therapeutic armamentarium for PNH, data remain scarce regarding the optimal selection, duration, and clinical indications for anticoagulation in patients receiving CIT. Moreover, the use of the newer class of DOACs has not been formally investigated.

in clinical trials; consequently, the choice of anticoagulants remains largely empirical, reflecting unstandardized institutional practices not based on robust scientific evidence [23–25].

This nationwide survey provides the first comprehensive overview of thrombosis management strategies in PNH across Italian Centers, encompassing more than half of the known national PNH population.

Recently, a large real-world study of 267 patients from 4 US Centers explored the features, predictors of TE, and anticoagulation strategies covering a total of 2,043 patient-years [23] Overall, 21% of patients developed TEs, 43% of which occurred at disease onset. The rate of TEs was halved in patients receiving CIT compared to untreated patients (20 vs. 40 TEs per 1,000 patient-years, respectively). This study described in detail the management of TEs, and the anticoagulation strategy consisting of warfarin (45%), DOACs (41%), and LMWH (14%), with a median treatment duration of 46 months. However, no information was specifically reported on the PAP and SAP strategies employed in the 4 US Centers, particularly in patients awaiting or on CIT.

The results of our survey reveal substantial variability in clinical practices, reflecting the lack of unified guidelines and confirming that PAP remains a controversial area. In fact, we observed that before CIT, only one-third of centers systematically prescribe prophylaxis in untreated patients or those awaiting complement inhibitors. Another quarter implement PAP selectively, based on thrombophilia screening, while a small minority base decision on the percentage of PNH neutrophils (9%). This diversity in clinical practice reflects both the absence of definitive evidence-based guidance and the historical perception of thromboembolism as a key driver of morbidity and mortality in untreated PNH [4–6, 11, 12]. Such clinical uncertainty in the field, has been emphasized by Fattizzo et al. [26], who highlighted the paucity of robust evidence supporting universal primary prophylaxis in PNH and underscored the importance of individualized risk assessment strategies, including the size of the PNH granulocyte population and the concomitant thrombophilia traits. Also Gurnari et al. have proposed that an extended coagulation workup at diagnosis may help identify a subset of patients who could benefit from early intervention [23]. However, uncertainty regarding this approach emerged in our survey, as only 11 out of 24 Centers perform thrombophilia screening at diagnosis as a potential tool to guide decisions on PAP treatment.

Among centers prescribing PAP, traditional anticoagulants were favored; vitamin K antagonists (VKAs) were utilized slightly more frequently than low molecular weight heparin (LMWH). The limited adoption of DOACs for primary prophylaxis may be attributed to concerns regarding their safety and efficacy in the setting of intravascular hemolysis and complement-mediated coagulopathy, reinforced by the lack of clinical trials investigating DOACs within this specific patient population.

The discontinuation of PAP represents another controversial issue, even though the initiation of CIT, good disease control (using LDH value < 1.5 the upper limit of normal as a surrogate marker for suppressed intravascular hemolysis), and the absence of signs of thrombosis may be considered reasonable criteria for the safe suspension of antithrombotic therapy. In our survey fifteen of sixteen centers (93%) that employed PAP reported discontinuing anticoagulation therapy following CIT initiation. Only one center continued PAP indefinitely in patients with underlying thrombophilia. This high degree of consensus suggests that clinicians perceive CIT as sufficiently protective against thrombosis to permit de-escalation of prophylactic anticoagulation in PNH patients without additional risk factors. This clinical behavior aligns with the established evidence that CIT substantially reduces thrombotic risk in PNH [8, 10].

Regarding SAP, our findings indicate that most centers continue SAP indefinitely irrespective of CIT. This practice reflects the scarcity of published cases on SAP discontinuation in patients receiving CIT [23, 27, 28]. Moreover, the reluctance to stop SAP is consistent with the conservative approach proposed by Brodsky who recommends a cautious approach to stopping anticoagulation due to the unpredictable nature of thrombosis in PNH, even in the setting of CIT [28]. He suggests evaluating SAP discontinuation when thrombotic symptoms have resolved and hemolytic activity is well controlled; such decisions must involve a careful discussion with the patient, emphasizing the paucity of evidence regarding the risks and benefits and taking into account patient preferences. However, in the last years, emerging data from long-term follow-up (up to 8 years) suggest that SAP discontinuation may be safe, without an increased risk of recurrent thromboembolic events, in a selected subset of patients with well-controlled hemolysis and no radiologic evidence of residual thrombosis [23].

Consistent with previous reports, the small number of centers in our survey, that discontinue SAP, take into consideration prolonged remission of TE, resolution of imaging findings (e.g., CT scan, MR angiography, or Doppler ultrasound), and sustained control of hemolysis.

However, in the absence of established guidelines, the reluctance of clinicians to discontinue SAP in patients with a prior history of TE is understandable, even when adequate disease control is achieved through CIT.

Furthermore, even in the SAP setting, DOACs were used by only a minority of centers, suggesting a persistent caution in adopting these newer anticoagulants in PNH, despite their expanding use in other high-risk thrombophilic states [19, 21]. While, preliminary case series and pharmacologic analyses suggest that DOACs may be safe and effective in selected PNH patients [23–25], robust prospective controlled trials trial remain lacking.

The management of breakthrough hemolysis (BTH) also shows significant variation in centers’ policy. LMWH initiation during BTH was influenced by the presence of precipitating factors (e.g., infections or immobilization) and the severity of hemolysis, highlighting a reactive management strategy rather than the application of standardized protocols. Fattizzo et al. have emphasized that not all BTH events carry the same thrombotic risk, and that clinical context—including markers such as LDH surge and D-dimer elevation— should guide the choice and introduction of anticoagulant therapy [29].

This study has several limitations including its reliance on self-reported institutional policies and incomplete data regarding individual patient characteristics. Nevertheless, the high response rate and broad national coverage strengthen the representativeness of our findings, which clearly suggest the urgent need to produce shared guidelines with the aim of harmonizing patient management in clinical practice. Moreover, a potential limitation of this study is the disparity in patient numbers across participating sites. However, our primary objective was to capture a comprehensive overview of real-world clinical practices in a setting where established guidelines are currently lacking. While we acknowledge that institutional volume and experience may influence clinical decision-making, this survey was not specifically powered to stratify results by center volume. Future large-scale international studies are warranted to investigate whether practice patterns significantly differ between high- and low-volume centers.”

In conclusion, the therapeutic landscape of PNH has been dramatically transformed by complement inhibitors, but clinical practice is not yet standardized. The findings of our study underscore the need for collaborative prospective studies, registry-based analyses and evidence-based recommendations to harmonize anticoagulation strategies for both primary (PAP) and secondary long-term (SAP) prophylaxis. However, given the rarity of the disease and the great variability of risks and manifestations of TEs, the conduct of traditional clinical trials remains inherently challenging. Therefore, guidelines derived from formal and structured expert consensus studies may represent a valuable tool to harmonize antithrombotic strategies and, ultimately, to improve the outcomes for patients with PNH.

## Data Availability

The datasets generated and analyzed during the current study are available from the corresponding author on reasonable request.
